# Photosynthetic response to increased irradiance correlates to variation in transcriptional response of lipid‐remodeling and heat‐shock genes

**DOI:** 10.1002/pld3.69

**Published:** 2018-07-10

**Authors:** Roxanne van Rooijen, Jeremy Harbinson, Mark G. M. Aarts

**Affiliations:** ^1^ Laboratory of Genetics Wageningen University and Research Wageningen The Netherlands; ^2^ Horticulture and Product Physiology Wageningen University and Research Wageningen The Netherlands; ^3^ Present address: Cluster of Excellence on Plant Science Heinrich Heine University Düsseldorf Germany

**Keywords:** *Arabidopsis thaliana*, high light stress, microarray, natural genetic variation, photosynthesis efficiency, photosystem II

## Abstract

Plants have evolved several mechanisms for sensing increased irradiance, involving signal perception by photoreceptors (cryptochromes), and subsequent biochemical (reactive oxygen species, ROS) and metabolic clues to transmit the signals. This results in the increased expression of heat‐shock response genes and of the transcription factor LONG HYPOCOTYL 5 (HY5, mediated by the cryptochrome photoreceptor 1, CRY1). Here, we show the existence of another response pathway in Arabidopsis. This pathway evokes the SPX1‐mediated expression activation of the transcription factor PHR1 and leads to the expression of several galactolipid biosynthesis genes. Gene expression analysis of accessions Col‐0, Ga‐0, and Ts‐1, showed activated expression of the SPX1/PHR1‐mediated gene expression activation pathway acting on galactolipids biosynthesis genes in both Ga‐0 and Col‐0, but not in Ts‐1. The activation of the SPX1/PHR1‐mediated response pathway can be associated with lower photosynthesis efficiency in Ts‐1, compared to Col‐0 and Ga‐0. Besides the accession‐associated activation of the SPX1/PHR1‐mediated response pathway, comparing gene expression in the accessions showed stronger activation of several heat responsive genes in Ga‐0, and the opposite in Ts‐1, when compared to Col‐0, in line with the differences in their efficiency of photosynthesis. We conclude that natural variation in activation of both heat responsive genes and of galactolipids biosynthesis genes contribute to the variation in photosynthesis efficiency in response to irradiance increase.

## 1 INTRODUCTION

The light‐use efficiency of photosynthesis is the result of the molecular, structural, and physiological state of the plant (Eberhard, Finazzi, & Wollman, 2008; Foyer, Neukermans, Queval, Noctor, & Harbinson, 2012; Zhu, Long, & Ort, 2008), which depends on many environmental factors. One of these, the level of irradiance, has a direct relation with photosynthesis light‐use efficiency as irradiance is the driving force for photosynthesis. At low irradiances, photosynthesis is fully light‐limited and photosynthetic light‐use efficiency is maximal. Beyond light‐limitation, light‐use efficiency decreases with increasing irradiances, resulting in the overall phenomenon of light‐saturation of photosynthesis at elevated irradiances (Long, Humphries, & Falkowski, 1994; Sinclair & Muchow, 1999). The decrease in light‐use efficiency from its maximum under wholly light‐limiting conditions implies that irradiance exceeds the capacity for photosynthetic metabolism (Long et al., 1994). A consequence of increased irradiance is an increase in the rate of damaging side reactions of photosynthesis that occur as a result of the reactive nature of many intermediates formed. These reactive intermediates come from redox signals mediated via changes in the degree of thioredoxin and plastoquinone reduction and increased formation of reactive oxygen species (ROS) (Vass, 2012). Reactive oxygen species (singlet oxygen [^1^O_2_], superoxide [$O_{2}^{-}$], the hydroxyl radical [•OH] and hydrogen peroxide [H_2_O_2_]) are the most conspicuously damaging by‐products of photosynthesis (Asada, 2006; Vass, 2012). The regulatory responses of photosynthesis, which are more active during stress (for example q_E_‐type nonphotochemical quenching or down‐regulation of electron transport at the level of the cytochrome b_6_/f complex) appear to reduce the formation of ROS, especially under high growth irradiances (Scheibe, Backhausen, Emmerlich, & Holtgrefe, 2005; Suzuki, Koussevitzky, Mittler, & Miller, 2012). The state of the photosynthesis apparatus – its composition, organization, and regulation – is thus under complex control. An increase in irradiance leading to excess will initially provoke rapid, physiological responses, including q_E_ type quenching, down‐regulation of electron transport at the cytochrome b_6_/f complex and increased CO_2_ fixation activity (Demmig‐Adams & Adams, 1992; Li, Wakao, Fischer, & Niyogi, 2009; Niyogi, 1999). If persistent, the increased irradiance will result in longer term acclimation of the photosynthetic apparatus, which can be detected at transcript and protein levels (Li et al., 2009; Walters, 2005). Different species and genotypes display different capacities to acclimate their photosynthetic apparatus to an irradiance increase so it is reasonable to infer that this is at least partly genetically determined (van Rooijen, Aarts, & Harbinson, 2015; van Rooijen et al., 2017).

In the rapid physiological regulatory response to irradiance increase, two major biochemical signals are known to be involved: a pH‐change within the chloroplast lumen, occurring within milliseconds after the irradiance increase; and redox changes, acting via changes in reduced thioredoxin levels, oxidized/reduced plastoquinone ratios, and a build‐up of ROS. These two classes of processes interact so changes in lumen pH regulation can affect the extent of redox changes and vice versa. Increases in ROS not only result from the irradiance increase itself but also from the concomitant temperature increase (Apel & Hirt, 2004). This combined irradiance and temperature effect causes the activation of heat‐shock factors, initiating further acclimation responses (Jung et al., 2013).

The transcriptome changes provoked by an increase in irradiance are not easy to interpret, being partly caused by the temperature increase rather than the irradiance increase (Swindell, Huebner, & Weber, 2007). Increased irradiance and increased temperature together induce a stress response at the site of photosynthesis, the chloroplast, mediated through a biochemical and molecular signaling cascade going from the chloroplast to the nucleus, known as retrograde signaling (Dietz, Wesemann, Wegener, & Seidel, 2018; Fey et al., 2005; Kleine & Leister, 2016). The main retrograde signals in response to increased temperature are the expression of heat‐shock proteins through the generation of ROS originating from the chloroplast, which is also thought to be a major retrograde signal required for the photosynthetic acclimation response to excess light (Jung et al., 2013; Rossel, Wilson, & Pogson, 2002; Vanderauwera et al., 2005). The retrograde signaling function of singlet oxygen and H_2_O_2_ has been largely unraveled (Dietz et al., 2018). Singlet oxygen and H_2_O_2_ modulate the activity of redox‐sensitive components of metabolic pathways such as the HDS‐MEcPP (HDS = HYDROXY‐2‐METHYL‐2‐(E)‐BUTENYL 4‐DIPHOSPHATE (HMBPP) SYNTHASE; MEcPP = 2‐C‐Methyl‐D‐erythritol‐2,4‐cyclodiphosphate) and the SAL1‐PAP (SAL1 is an enzyme with 3′(2′), 5′‐biphosphate nucleotidase and inositol polyphosphate 1‐phosphatase activities; PAP= 3′‐phosphoadenosine 5′‐phosphate) pathways. Both pathways lead to posttranscriptional gene silencing in the nuclear genome (Estavillo et al., 2011; Xiao et al., 2012). Besides modulating transcriptional networks, singlet oxygen and H_2_O_2_ also modulate translation of transcripts via the inhibition of conserved TARGET OF RAPAMYCIN COMPLEX (TORC) serine/threonine kinases and the simultaneous activation of SUCROSE NONFERMENTING‐RELATED KINASE 1 (SnRK1), which regulate the balance between energy status and growth regulation (Robaglia, Thomas, & Meyer, 2012). Another regulatory mechanism is the ROS‐mediated control of mRNA stability through the formation of stress granules and processing (p‐)bodies (Dietz et al., 2018; Uniacke & Zerges, 2008).

In addition to the indirect detection of increase in irradiance through increased temperature and the formation of ROS, plants directly detect increases in irradiance through their cryptochrome photoreceptors (Kleine, Kindgren, Benedict, Hendrickson, & Strand, 2007). Of the two plant cryptochromes in *Arabidopsis thaliana* (Arabidopsis), CRY1 and CRY2, only CRY1 initiates a transcriptional response to increased irradiance, mediated by the transcription factor LONG HYPOCOTYL 5 (HY5) (Kleine et al., 2007; Lee et al., 2007). The gene expression response to increased irradiance initiated by the heat‐shock factors and the response initiated via the HY5 transcription factor are considered to be two distinct gene activation pathways, both in function and in time (Yamamoto et al., 2004). However, it was recently unraveled that HY5 also negatively controls the unfolded protein response in Arabidopsis, in which a set of molecular chaperones is expressed to assist the endoplasmic reticulum in protein folding during stress situations (Nawkar et al., 2017).

As a last indirect sensing mechanism, plants sense increased irradiance through an accumulation of the phytohormone abscisic acid (ABA) in leaves (Galvez‐Valdivieso et al., 2009). ABA, high light, and oxidative stress similarly down‐regulate *Lhcb* genes; hydrogen peroxide produced in chloroplasts under high light conditions interacts with the ABA signaling network to regulate *Lhcb* expression (Borisova‐Mubarakshina et al., 2015; Staneloni, Rodriguez‐Batiller, & Casal, 2008).

Until now, the response of Arabidopsis to increased irradiance has only been studied in one accession, Col‐0. In our study we include two additional, natural, accessions of Arabidopsis, with contrasting photosynthesis responses to increased irradiance (van Rooijen et al., 2015), to reveal common, as well as genotype‐specific, transcriptional responses associated with the acclimation of photosynthesis efficiency. Analysis of different time‐points within one study allows the detection of transient expression patterns throughout the acclimation response. Next to the induction of genes involved in the protection against a temperature increase, and genes involved in the generation and perception of ROS ‐ mediated through (retrograde) signaling ‐ we expect the increased irradiance to temporarily arrest plant growth, which should lead to a decrease in the expression of genes involved in growth and development. In the course of acclimation, expression of these genes is expected to return to the status before the increase in irradiance.

## 2 MATERIALS AND METHODS

### 2.1 Plant material and growth conditions

The Arabidopsis accessions Columbia‐0 (Col‐0, CS76113), Gabelstein‐0 (Ga‐0, CS76133) and Tossa de Mar‐1 (Ts‐1, CS76268) were grown as previously described (van Rooijen et al., 2015). In short, the plants were grown under controlled conditions in the Phenovator (Flood et al., 2016), on rockwool, automatically watered with nutrient solution, in a 10 hr/14 hr day/night cycle, with the temperature set at 20/18°C (day/night), and relative humidity set at 70%. CO_2_ levels were ambient. Under controlled conditions, plants in the light were kept at a constant, low, irradiance of 100 μmol m^−2^ s^−1^ (Philips 610 fluorescent tubes, MASTER TL5 HO, 80W). In the increased irradiance treatment, the irradiance was increased to 550 μmol m^−2^ s^−1^ at the onset of the photoperiod on the 25th day after sowing. In the increased temperature treatment, the irradiance was kept at 100 μmol m^−2^ s^−1^, but the temperature was increased from 20°C to 30°C during the day. Photosynthesis efficiency was measured as previously described (van Rooijen et al., 2015, 2017).

### 2.2 RNA sample preparation

For all three genotypes, rosettes of nine plants per genotype were harvested on the day of the irradiance increase, 1 hr after the start of the photoperiod and flash‐frozen in liquid nitrogen. For the Col‐0 accession, nine rosettes were additionally harvested on the day of the irradiance increase 3.5 hr after the start of the photoperiod, and again 1 day later, at 1 hr after the start of the photoperiod, so 25 hr after increasing the irradiance to 550 μmol m^−2^ s^−1^. As a control, for all three genotypes rosettes of nine plants per genotype that were kept in low light (100 μmol m^−2^ s^−1^) were harvested at 1 hr after the start of the photoperiod, and for Col‐0 also at the additional time points, at 3.5 and 25 hr. The nine rosettes were subdivided in three groups of three rosettes and treated as three biological replicates. For each biological replicate for each accession‐treatment combination, the three rosettes were pooled as one sample for RNA isolation.

Total RNA was extracted using the Direct‐zol RNA mini‐prep kit from Zymo Research (www.zymoresearch.com). The RNA quality control, labeling, microarray hybridization, and data extraction were performed at ServiceXS B.V. (part of GenomeScan B.V., Leiden, The Netherlands). The RNA concentration was measured using a DropSense96 spectrophotometer (Trinean N.V., Gentbrugge, Belgium). The RNA quality and integrity was determined using Lab‐on‐Chip analysis on the Agilent 2100 BioAnalyzer (Agilent TEchn9ologies, Inc., Santa Clara, CA, USA). Single‐strand‐cDNA (ss‐cDNA) was prepared using the Ovation^®^ PicoSL WTA System V2 (NuGEN Technologies, Inc., San Carlos, CA, USA) according to the manufacturer's specifications, with an input of 50 ng total RNA. Labeling and fragmentation of the ss‐cDNA was performed with the Encore™ Biotin Module (NuGEN Technologies, Inc., San Carlos, CA, USA).

### 2.3 Microarray hybridization, scanning, and data analysis

Per sample, 2.5 μg of the labeled ss‐cDNA was hybridized onto an AraGene 1.1ST Array plate (Affymetrix, Santa Clara, CA, USA). Hybridization and scanning was performed on a GeneTitan (Affymetrix, Santa Clara, CA, USA). Image analysis and extraction of raw expression data was performed with the Affymetrix Expression Console™ v1.2.1. with “Gene‐level Default: RMA‐Sketch” settings. Raw data were analysed using the Bioconductor packages in the statistical programming language R (http://www.r-project.org) (Gentleman et al., 2004). The Affymetrix AraGene 1.1ST microarray oligonucleotide probe annotation list TAIRT v19 cdf file was downloaded from Brainarray (http://brainarray.mbni.med.umich.edu/Brainarray/Database/CustomCDF/19.0.0/tairt.asp) and gene expression was normalized using the Robust Multi‐array Average (RMA) algorithm (Irizarry et al., 2003), after which a linear model was fitted for every gene. The empirical Bayes method was used to determine significant differences between the samples and the Benjamini and Hochberg method was used for adjustment of the *p* values for multiple testing (Benjamini & Hochberg, 1995; Efron & Tibshirani, 2002). The limma package was used to conduct log2 fold changes between control (low light, LL) conditions and experimental (high light, HL) conditions. Genes were selected with log2 fold change ≤0.7, or with log2 fold change ≥1.4. The limma package was also used to determine differential responsiveness of genes between accessions or between time points with a *p*‐value cut‐off of 0.05. The microarray dataset has been deposited at NCBI with accession number GSE115051, and can be accessed through http://www.ncbi.nlm.nih.gov/geo/query/acc.cgi?acc=GSE115051.

### 2.4 Quantitative reverse transcription PCR (qRT‐PCR)

In a separate experiment, but with identical experimental set‐up as used for the microarray experiment, nine rosettes were similarly harvested, on the 25th day after sowing, for all accession‐treatment combinations at 1 and 3.5 hr after the start of the photoperiod, and immediately frozen in liquid nitrogen. As a control, rosettes of nine plants per accession that were grown in low light (100 μmol m^−2^ s^−1^) were harvested at the same time points. Three rosettes for each accession‐treatment combination were pooled as one sample for RNA isolation, in three biological replications. RNA was extracted according to Onate‐Sánchez and Vicente‐Carbajosa (2008). After normalization of RNA concentrations, cDNA was synthesized using the Iscript cDNA synthesis kit from Bio‐RAD (www.bio-rad.com). Quantitative real‐time reverse transcriptase PCR (qRT‐PCR) was performed with three technical replicates for each biological replicate using the SYBR‐green mastermix from Bio‐RAD. Three reference genes were used for normalization of samples: *UBIQUITIN7* (*UBQ7,* At2 g35635), *CYTOCHROME B5 ISOFORM E* (*CB5E,* At5 g53560), and *UBIQUITIN THIOESTERASE* (At1 g28120); expression levels of *UBQ7* and *CB5E* were previously found to be constant under excess light (Jung et al., 2013; Wunder et al., 2013), and expression levels of *UBIQUITIN THIOESTERASE* were previously found to be stable in several genome‐wide expression studies involving irradiance changes (Genevestigator database (Hruz et al., 2011), https://genevestigator.com/gv/). The primers used for qRT‐PCR are listed in Supporting Information Table S7.

## 3 RESULTS

### 3.1 Heat‐shock response and RNA binding protein genes are consistently responding to an increase in irradiance in Arabidopsis

**Figure 1:**
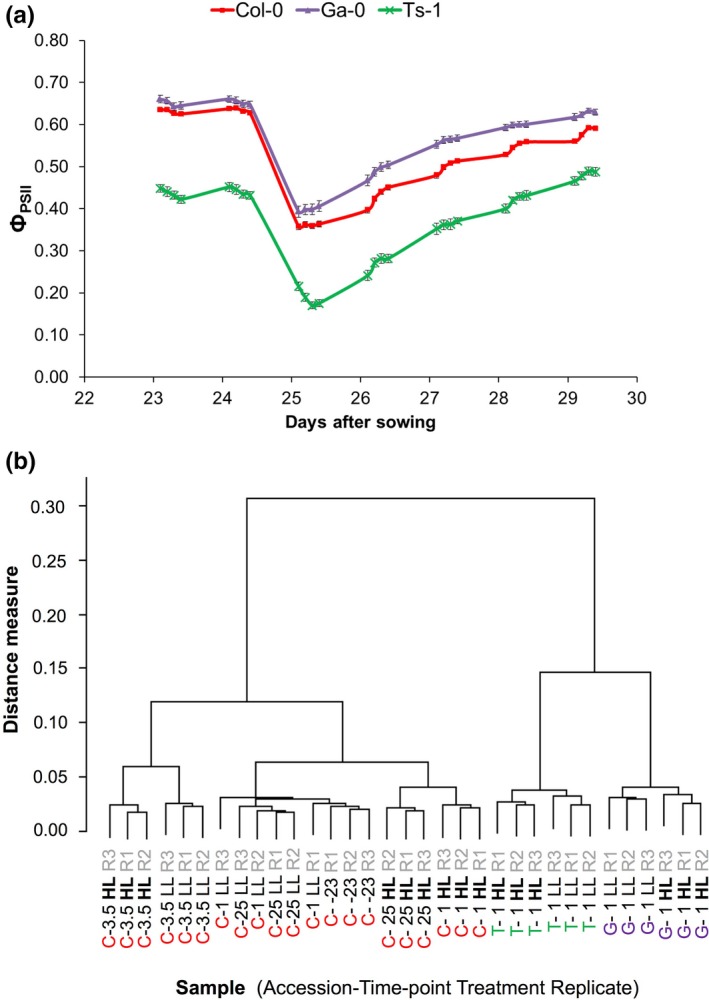
The effect of genotype on the photosynthetic response over time to increased irradiance in Arabidopsis. (a) Representative photosynthetic (Ф_PSII_) phenotypes for Arabidopsis accessions Col‐0, Ga‐0, and Ts‐1, grown for 24 days in 100 μmol m^−2^ s^−1^ growth irradiance and subsequently 6 days in 550 μmol m^−2^ s^−1^ growth irradiance, measured from day 23 until day 30 after sowing, at four time‐points per day; (b) Dendrogram of Pearson distance measures, based on microarray‐based transcriptome data representing gene expression in rosettes of Arabidopsis accessions Col‐0, Ts‐1 and Ga‐0 exposed to low light (LL; 100 μmol m^−2^ s^−1^ growth irradiance) control conditions or increased irradiance (high light, HL; 550 μmol m^−2^ s^−1^ growth irradiance), sampled at 23 hr before the irradiance increase (−23), and 1, 3.5 and 25 hr after the irradiance increase (1, 3.5, 25), in three experimental replicates (R1, R2 and R3)

Arabidopsis accessions Col‐0, Ga‐0 and Ts‐1 were grown for 24 days under 100 μmol m^−2^ s^−1^ growth irradiance (low light, LL), and from the next day onwards exposed to an irradiance of 550 μmol m^−2 ^s^−1^ (high light, HL), which is saturating for photosynthesis in these low‐light grown plants (van Rooijen et al., 2015). Upon exposure to increased irradiance, the accessions display different photosynthetic light‐use efficiencies (determined by measuring Φ_PSII_, the light‐use efficiency of photosystem II (PSII) electron transport, also known as Fq′/Fm′) (Figure 1a). Ts‐1 has a generally lower Φ_PSII_ than Col‐0 and Ga‐0, independent of the growth irradiance level. After the switch from low light to high light, all accessions show a decrease in Φ_PSII_ with Ga‐0 showing the smallest drop and highest recovery. Ts‐1 recovers most, reaching the same level of Φ_PSII_ as it had before the irradiance increase after 4 days, which was not achieved by the other two accessions. Col‐0 Φ_PSII_ recovers most during the day, whereas in Ga‐0 it recovers most during the night. These accession‐specific differences in Φ_PSII_ response are reflected in measurements of the chlorophyll reflectance index and the photochemical reflectance index (Supporting Information Figure S1) (Datt, 1999; Gamon, Serrano, & Surfus, 1997). Indirectly related to photosynthetic light‐use efficiency is the anthocyanin reflectance index, a proxy for anthocyanin content (Gitelson, Merzlyak, & Chivkunova, 2001). Also the anthocyanin reflectance index shows accession‐specific differences (Supporting Information Figure S1). However, we did not find an accession‐correlated relation between anthocyanin reflectance index and photosynthetic light use efficiency. A rosette transcriptome analysis was performed on the accessions Ts‐1, Col‐0, and Ga‐0 to identify common and accession‐specific responsive genes, at 1 hr after the lights were switched on at HL. For Col‐0, a time‐series transcriptome analysis was performed to investigate the temporal effect on gene expression.

**Figure 2:**
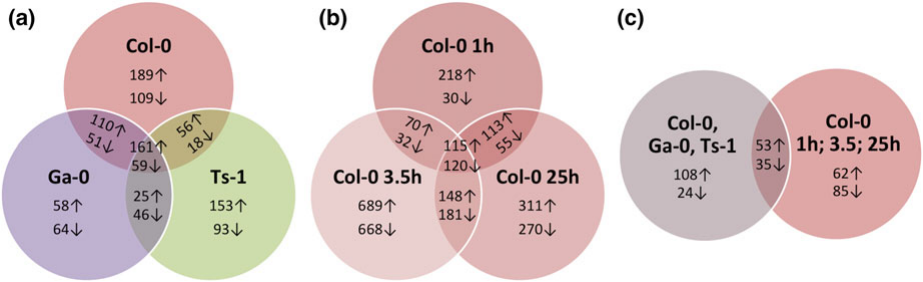
Arabidopsis accession‐ and time‐specific gene expression responses to increased irradiance. Venn diagrams displaying the number of significantly (*p* = 0.05) differentially (more than 1.4‐fold up‐ or down‐regulated) expressed genes when comparing (a) rosettes of plants grown at low light (100 μmol m^−2^ s^−1^ growth irradiance) of three accessions (Col‐0, Ga‐0, or Ts‐1; control) to those of plants exposed for the first day to high light (550 μmol m^−2^ s^−1^ growth irradiance), 1 hr after lights were switched on; and (b) rosettes of control Col‐0 plants grown at low light (100 μmol m^−2^ s^−1^ growth irradiance) at 1, 3.5 or 25 hr after lights on, to those of Col‐0 plants at 1, 3.5, or 25 hr after exposure to increased irradiance (550 μmol m^−2^ s^−1^ growth irradiance); and (c) the differentially expressed genes shared by all accessions indicated in (a) with those shared by all time‐points indicated in (b). Arrows indicate up‐ and down‐regulation compared to controls

Hierarchical clustering distinguishes all treatments, except for the Col‐0 LL control treatments at three consecutive days (1 hr after the lights were switched on), which treatments were all very similar (Figure 1b). The accession differences are most prominent and the effect of time seems to be larger than the effect of the HL treatment, suggesting that the LL‐HL comparison will provide a specific set of HL‐responsive genes and a general set of stress‐responsive genes. Genes that were statistically significantly more than 1.4‐fold up‐ or down‐regulated when comparing the increased irradiance treatment with the control treatment, will be further refer to as “responsive genes” (Figure 2).

Over all, a total of 752 genes were more than 1.4‐fold up‐regulated, and 440 genes were more than 1.4‐fold down‐regulated in at least one of the three accessions 1 hr after the irradiance increase from 100 to 550 μmol m^−2^ s^−1^, when compared to untreated plants (Figure 2a). Of these responsive genes, 161 up‐regulated and 59 down‐regulated genes were shared among all three accessions (Figure 2a). In Col‐0, a total of 1,664 genes were more than 1.4 fold up‐regulated, and 1,356 genes were more than 1.4‐fold down‐regulated at any of the three time‐points after the switch, of which 115 were up‐regulated at all three time‐points, and 120 were down‐regulated at all three time‐points (Figure 2b). The genes common to both comparisons (i.e. responding in all accessions and at all time‐points in Col‐0) were considered to represent a core set of 88 Arabidopsis genes (53 up‐regulated, and 35 down‐regulated) that respond to an irradiance increase (Supporting Information Tables S1 and S2).

**Figure 3:**
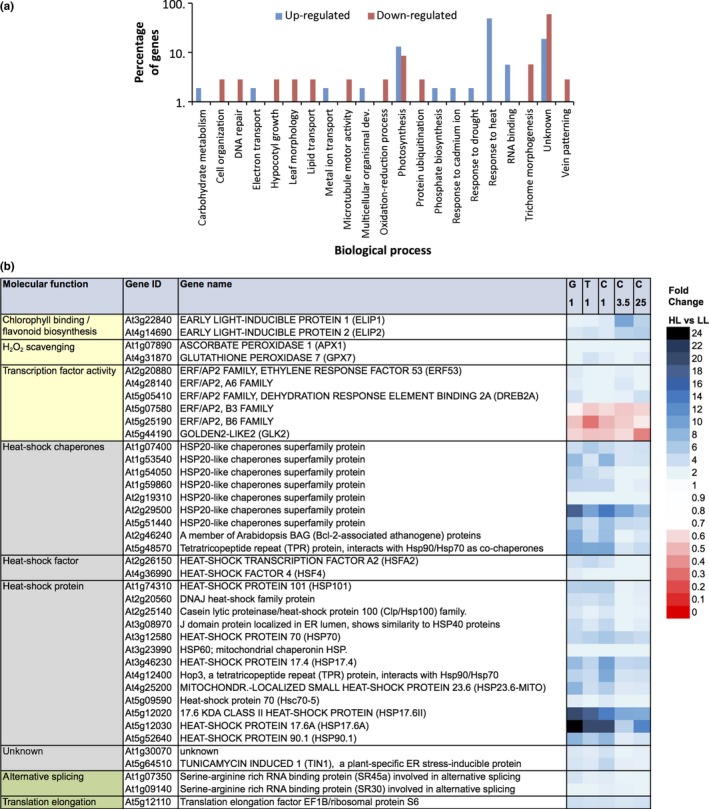
Gene ontology and functional annotation of a core set of Arabidopsis genes expressed in rosettes, showing a transcriptional response upon exposure to increased irradiance in all three tested accessions (Ga‐0, Ts‐1 and Col‐0) and at all examined time‐points (after 1, 3.5 and 25 hr) when compared to plants grown in control conditions. (a) Gene ontology enrichment for biological process of Arabidopsis genes differentially expressed in rosettes at 1, 3.5, or 25 hr after exposure to increased irradiance, when comparing three accessions, Ga‐0, Ts‐1, and Col‐0 (G, T, C), and three time‐points (1, 3.5 and 25; in Col‐0 only) to untreated controls; (b) Heat map displaying fold changes in gene expression in increased irradiance (HL) conditions versus low irradiance (LL) control conditions, of core genes with biological functions in photosynthesis (yellow highlighted), response to heat (gray highlighted), and RNA binding (green highlighted), as indicated in (a). All listed genes were statistically significantly more than 1.4‐fold up‐ or down‐regulated at all time‐points and in all accessions when compared to control conditions. For the complete gene list see Supporting Information Tables S1 and S2

Gene ontology analysis regarding the biological processes associated with this core set of 88 genes shows that up‐regulated genes are enriched for involvement in “heat‐shock response”, “photosynthesis” and “RNA binding” processes; while the down‐regulated genes show only minor enrichment for “photosynthesis” processes (Figure 3a). When comparing HL and LL conditions the enriched biological processes comprise 39 differentially expressed genes (Figure 3b). The majority of these encode heat‐shock proteins (HSPs), which are up‐regulated. Only three photosynthesis‐associated genes are identified among the 39 differentially expressed genes; these three all encode transcription factors and are all down‐regulated.

Comparison of the fold‐changes of the core set of responsive genes between the three accessions (Ga‐0, Ts‐1, and Col‐0), or between the three time‐points (1, 3.5, and 25 hr after exposure to irradiance increase), revealed differences in responsiveness for some genes (Supporting Information Tables S1 and S2). Of these, only two differed in their expression when comparing accessions. These were the *DEHYDRATION RESPONSIVE ELEMENT BINDING 2A (DREB2A;* At5 g05410*)* gene, which belongs to the drought‐related ERF/AP2 transcription factor family, and the *SERINE‐ARGININE RICH RNA BINDING PROTEIN 45a (SR45a*; At1 g07350), which is involved in alternative RNA splicing. Twenty‐seven genes differed in a time‐point comparison, of which all were heat‐shock response genes. Nine core genes differed in both the interaccession and time‐point comparisons, and these comprised eight heat‐shock response genes and one DREB‐like drought‐related transcription factor other than DREB2A, belonging to the ERF/AP2 transcription factor family.

The expression of four core responsive genes showed a high correlation (*r *>* *0.5) with the photosynthesis efficiency of the accessions (measured as Φ_PSII_); these are the *CELL DIVISION CYCLE 48B* (*CDC48B*) gene, an AAA‐type ATPase (At3 g53230), the *HSP60* heat‐shock protein gene (At3 g23990), and the *SIAMESE‐RELATED 5* (*SMR5*) cell cycle inhibitor gene (At1 g07500; Supporting Information Table S1). The expression of four other genes was negatively correlated with photosynthesis efficiency (*r *<* *−0.5); *DREB2A* (At5 g05410), the *HSFA2* heat‐shock factor gene (At2 g26150), an *HSP20‐like* heat‐shock protein chaperone gene (At1 g07400), and the *MULTIPROTEIN BRIDGING FACTOR 1C* (*MBF1C*) heat‐responsive transcriptional coactivator gene (At3 g24500) (Supporting Information Table S1).

### 3.2 Heat‐shock response and lipid‐remodeling genes differ in response to high light when comparing accessions

Next to the shared, common responses, the three accessions also showed differential responses. 1 hr after the irradiance increase a total of 1,192 genes were more than 1.4‐fold up‐regulated or down‐regulated in at least one of the three accessions, when compared to untreated plants. Of these, 752 genes were up‐regulated, and 440 genes were down‐regulated (Figure 2a). Of the 1,192 responsive genes, 153 genes were identified as accession‐specific responsive genes (Supporting Information Tables S3 and S4), meaning they were differentially responsive (*p* = 0.05) between two or all three accessions (i.e. Col‐0, Ga‐0 and Ts‐1). Of these 153 genes, 22 genes were also differentially expressed between accessions irrespective of the irradiance level, while the others responded specifically differentially to the HL treatment (Supporting Information Tables S3 and S4). Of the 153 accession‐specific genes, 60 genes were positively correlated with photosynthesis efficiency (*r *>* *0.5) and 55 were negatively correlated (*r *<* *−0.5; Supporting Information Tables S3 and S4).

**Figure 4:**
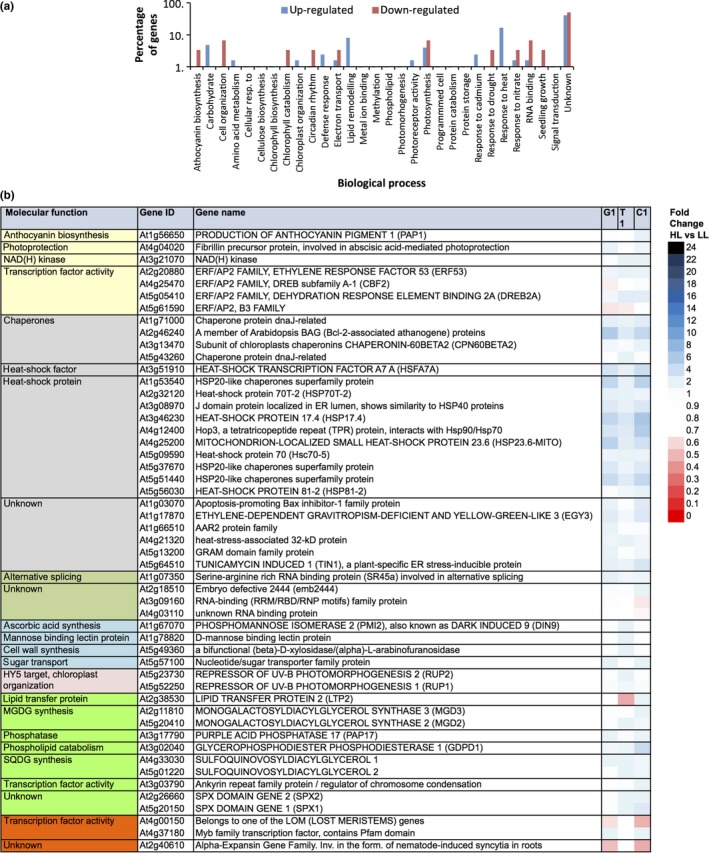
Gene ontology and functional annotation of Arabidopsis genes expressed in rosette leaves, showing an accession‐specific transcriptional response upon exposure to increased irradiance. (a) Gene ontology enrichment for biological process of Arabidopsis rosette‐expressed genes >1.4 fold differentially expressed at 1 hr after exposure to increased irradiance (HL) when compared to low irradiance (LL) plants (control), and differentially expressed between at least two of the three accessions Ga‐0, Ts‐1, and Col‐0; (b) Heat map presenting gene expression fold changes of genes differentially expressed at 1 hr after exposure to increased irradiance when compared to low irradiance, control, plants, and differentially expressed between at least two of the three tested accessions (G 1, T 1, C 1), annotated to be involved in one of the enriched biological processes as indicated in (a): photosynthesis (yellow highlight), response to heat (gray highlight), RNA binding (olive highlight), carbohydrate metabolism (blue highlight), photoreceptor activity (pink highlight), lipid‐remodeling (light‐green highlight), and cell organization (orange highlight). For the complete list of differentially expressed genes, see Supporting Information Tables S3 and S4

Gene ontology analysis of the 153 accession‐specific genes showed enrichment for “heat‐shock response”, “lipid‐remodeling”, “carbohydrate metabolism”, and “photosynthesis” among the up‐regulated genes; and for “cell organization”, “photosynthesis” and “RNA binding” among the down‐regulated genes (Figure 4a). Most prominent among the 51 genes classified in one of the enriched processes are the heat‐shock response genes (Figure 4b).

Remarkably, we could not confirm the up‐regulation of genes belonging to the well‐studied CRY1/HY5 pathway (Kleine et al., 2007). The up‐regulation of *HY5* was below the threshold we set in our micro array study; the average fold‐changes were: in Ga‐0 up‐regulated 1.3‐fold after 1 hr, in Ts‐1 up‐regulated 1.7‐fold after 1 hr, in Col‐0 up‐regulated 1.2‐fold after 1 hr, 1.5‐fold after 3.5 hr, and 0.8‐fold after 25 hr in this study. Although the up‐regulation in Ts‐1 was above our threshold of 1.4‐fold, it was not selected for the accession‐specific up‐regulated genes, because it was not statistically different from the fold‐change in Col‐0 and Ga‐0 (*p* = 0.05).

Additionally, we did not find up‐regulated gene clusters involved in ABA responsiveness in this study. Gene clusters involved in ABA responsiveness are known to be induced by high light with reduced infra‐red but repressed by high light containing infra‐red (Rossel et al., 2002). We probably did not find up‐regulation of this cluster in this study, because the ABA signaling network causes down‐regulation of *Lhcb* genes only after several days of high light treatment, and not during the first hours of high light treatment (Alsharafa et al., 2014).

### 3.3 Differential expression in genes for photosynthetic response and heat‐shock response when comparing sequential time‐points after increased irradiance in Col‐0

**Figure 5:**
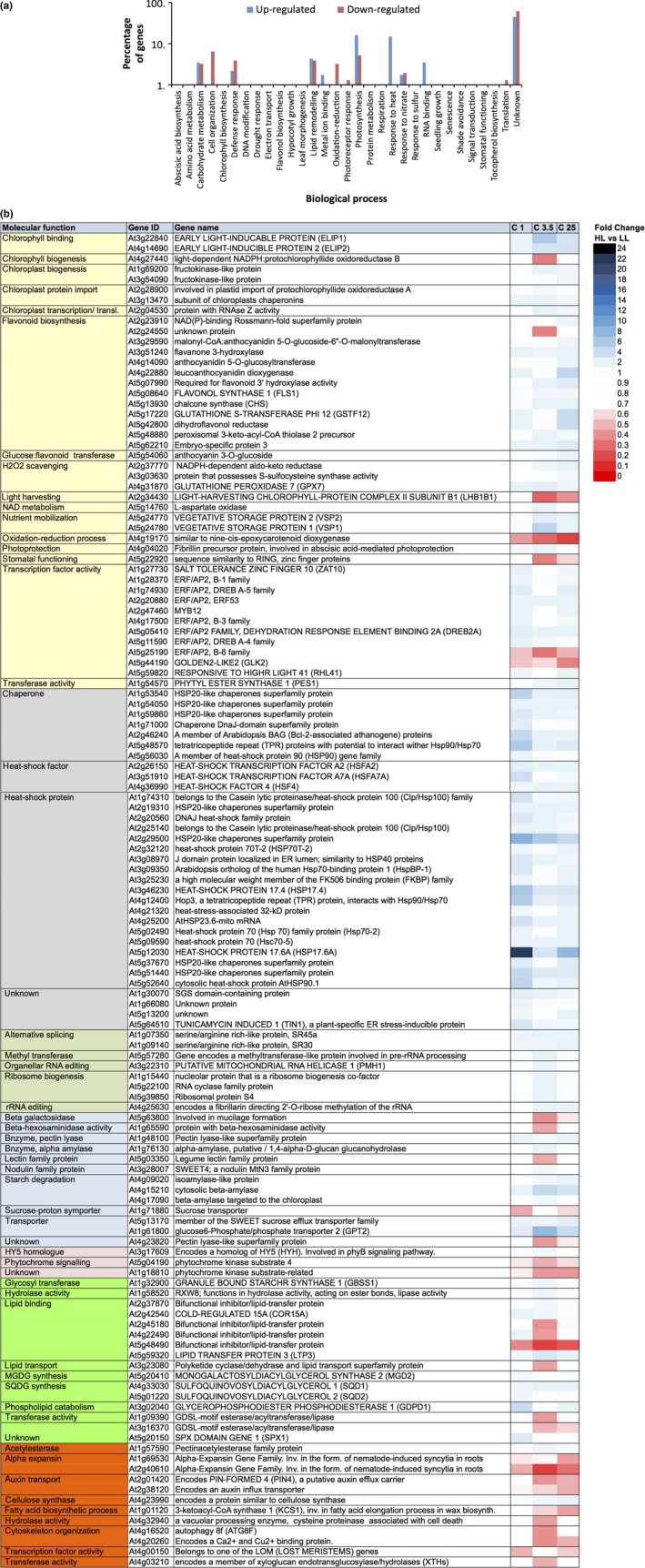
Gene ontology and functional annotation of Arabidopsis Col‐0 rosette genes showing time‐point‐specific transcriptional response upon exposure to increased irradiance. (a) Gene ontology enrichment for biological process of Arabidopsis Col‐0 rosette genes >2 fold differentially expressed after exposure to increased irradiance (HL) when compared to low irradiance (LL) plants (control), and differentially expressed on at least two of the three tested time‐points, 1, 3.5, and 25 hr after irradiance increase; (b) Heat map presenting gene expression fold changes of genes differentially expressed in Col‐0 after exposure to increased irradiance when compared to low irradiance, control, plants, and differentially expressed between at least two of the three tested time‐points (1, 3.5, 25 hr) annotated to be involved in one of the enriched biological processes as indicated in (a): photosynthesis (yellow highlight), response to heat (gray highlight), RNA binding (olive highlight), carbohydrate metabolism (blue highlight), photoreceptor activity (pink highlight), lipid‐remodeling (light‐green highlight), and cell organization (orange highlight). For the complete list of differentially expressed genes, see Supporting Information Tables S5 and S6

To identify transient expression patterns throughout the acclimation response, and to study the effect of diurnal rhythm on gene expression responses during longer term acclimation, time‐point‐specific expression responses were characterized in Col‐0 at three time‐points after the irradiance increase. When compared to untreated plants, a total of 3,020 genes were significantly more than 1.4‐fold up‐ or down‐regulated in at least one of the three time‐points after the irradiance increase (Figure 2b). When focusing on the 384 most responsive genes, 229 genes were more than 2.0‐fold up‐regulated and 155 genes more than 2.0‐fold down‐regulated (Supporting Information Tables S5 and S6). Gene ontology analysis of the 384 time‐point‐specific genes showed that those in the “photosynthesis”, “heat‐shock response”, “lipid‐remodeling”, “RNA binding”, and “carbohydrate metabolism” classes were enriched among the up‐regulated genes, while the classes “cell organization”, “photosynthesis”, and “lipid‐remodeling” were enriched among the down‐regulated genes (Figure 5a). Figure 5b lists the time‐point‐specific classification of the responsive genes based on their associated biological process, and their expression difference when compared to control plants. Of the 384 time‐specific genes, 58 genes correlated with an *r *>* *0.5 to the photosynthesis efficiency measured in the same accession in the same time points and 78 correlated with an *r *<* *−0.5 (Supporting Information Tables S5 and S6). The majority of the photosynthesis related time‐point specifically regulated genes in Col‐0 are the genes involved in flavonoid/anthocyanin biosynthesis as well as the transcription factor encoding genes belonging to the ERF/AP2 family.

### 3.4 Light response versus heat response

In order to distinguish between the direct effect on gene expression of an increase in irradiance and the indirect effect caused by the increase in temperature due to the increased irradiance, the expression of nine genes was determined using quantitative reverse transcriptase PCR (qRT‐PCR). In this set, genes were included that classified as being involved in any of the overrepresented biological processes (Figures 3, 4 and 5). Additionally, the genes were either highly differentially expressed or among the genes for which expression was correlated with Φ_PSII_. The plants were grown at either an increased irradiance, which also resulted in an increased temperature (HL), or an increased temperature (but with no increase in irradiance) (HT) and compared to plants grown under control conditions (no increased irradiance, no increased temperature; LL, LT). Plants were sampled 1 and 3.5 hr after the start of irradiance.

**Figure 6:**
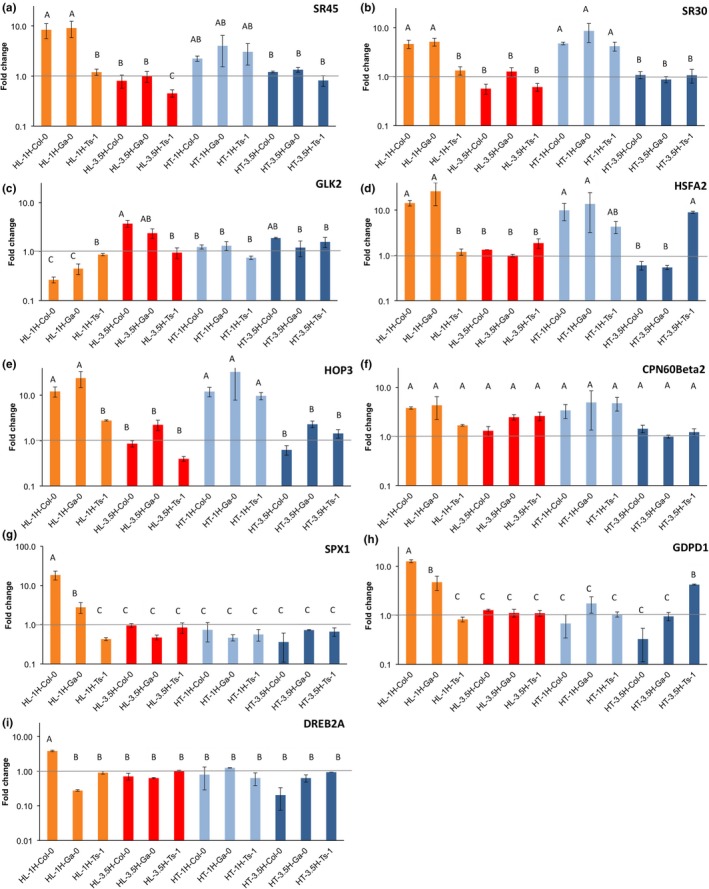
Gene expression changes in response to increased irradiance or in response to increased temperature, when compared to control conditions (no increased irradiance, no increased temperature). Gene expression differences, expressed as fold changes compared to control conditions (average ± *SE*), as determined by qRT‐PCR analysis of nine representative genes selected from Figures 3, 4, and 5, in Arabidopsis rosettes at 1 hr (1H; orange) and 3.5 hr (3.5H; red) after an irradiance increase from 100 to 550 μmol m^−2^ s^−1^ (HL), as well as 1 hr (light blue) and 3.5 hr (dark blue) after a temperature increase from 20 to 30°C (HT) in accessions Col‐0, Ga‐0 and Ts‐1. The control conditions were 100 μmol m^−2^ s^−1^ irradiance and 20°C temperature, with samples taken 1 hr after lights on. The following genes were analysed: (a) *SR45a* (At1 g07350); (b) *SR30* (At1 g09140); (c) *GLK2* (At5 g44190); (d) *HSFA2* (At2 g26150); (e) *HOP3* (At4 g12400); (f) *CPN60beta2* (At3 g13470); (g) *SPX1* (At5 g20150); (h) *GDPD1* (At3 g02040); (i) *DREB2A* (At5 g05410). Letters indicate statistically significant differences as determined by analysis of variance (*p* < 0.05)

We investigated in qRT‐PCR two genes involved in alternative splicing *SR45a* (At1 g07350) and *SR30* (At1 g09140); two transcription factors *GLK2* (At5 g44190) and *DREB2A* (At5 g05410), three heat‐shock genes *HSFA2* (At2 g26150), *HOP3* (At4 g12400), and *CPN60BETA2* (At3 g13470); and two genes involved in lipid‐remodeling *SPX1* (At5 g20150) and *GDPD1* (At3 g02040). Of these genes, all but *CPN60Beta2* were indeed confirmed to be responsive to increased irradiance (Figure 6), with *GLK2, SPX1, GDPD1,* and *DREB2A* to be specifically responsive to the irradiance increase and not to the temperature increase. Expression of *GLK2, SPX1, GDPD1,* and *DREB2A* was accession‐specific, with little or no response in Ts‐1. Also the four genes responsive to both the irradiance and temperature increases (*SR30, SR45a, HSFA2*, and *HOP3*) were accession‐specific, again with little or no response in Ts‐1. The accession‐specific effect was more pronounced after the irradiance increase than after the temperature increase (Figure 6).

## 4 DISCUSSION

**Figure 7:**
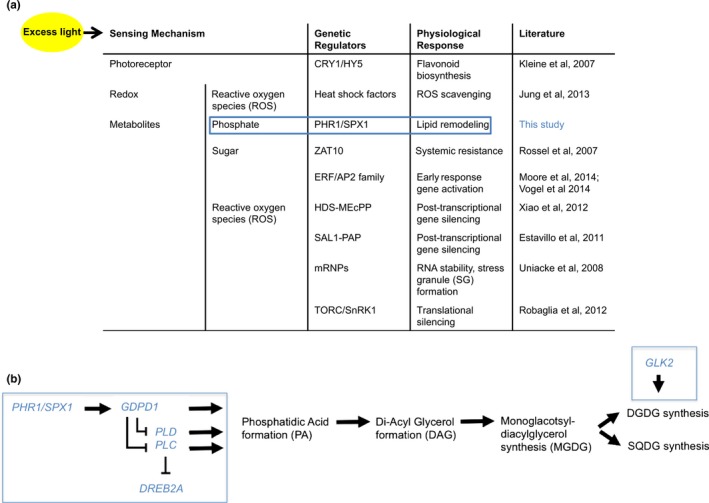
Summary table, incorporating lipid‐remodeling as an additional response pathway to the known signal transduction pathways leading to transcriptional responses upon increased irradiance (a); and a schematic model (b) representing the newly proposed lipid‐remodeling signal transduction pathway. Several genes of this pathway (*SPX1, GDPD1, DREB2A, GLK2*; in blue boxes) were found to be up‐regulated in response to increased irradiance. The processes they influence are indicated in black. CRY1 = CRYPTOCHROME 1; HY5 = LONG HYPOCOTYL 5; PHR1 = PHOSPHATE STARVATION RESPONSE 1; ZAT10 = SALT TOLERANCE ZINC FINGER 10; ERF/AP2 = ETHYLENE RESPONSE FACTOR/APETALA2; SPX1 = SYG1, PHO81, AND XPR1 HOMOLOGUE 1; GDPD1 = GLYCEROPHOSPHO‐DIESTER‐PHOSPHO‐DIESTERASE1; PLD = PHOSPHOLIPASE D; PLC = PHOSPHOLIPASE C; DREB2A = DEHYDRATION RESPONSE ELEMENT BINDING PROTEIN 2A; DGDG = DIGALACTOSYL DIACYLGLYCEROL; SQDG = SULFOQUINOVOSYL DIACYLGLYCEROL; GLK2 = GOLDEN‐LIKE 2

Different natural accessions of Arabidopsis vary in their acclimation of photosynthetic efficiency (measured as Ф_PSII_) to an increase in growth irradiance (van Rooijen et al., 2015, 2017). This study has been designed to identify transcriptional response pathways that are associated with natural variation in Ф_PSII_ acclimation to an increase in growth irradiance between three Arabidopsis accessions. Average levels of growth irradiance is sensed by the plant's nuclear‐encoded photoreceptors (phytochromes) to induce appropriate photomorphogenic development, but at high levels the irradiance is sensed through plastid‐localized retrograde signaling pathways (Martin et al., 2016). This study confirms some of the known transcriptional response pathways to increased irradiance (Figure 7a), and it identifies a transcriptional response pathway involved in lipid‐remodeling to be up‐regulated after irradiance increase.

### 4.1 Expression variation in lipid‐remodeling genes associates with natural variation in photosynthesis response to increased irradiance

Gene ontology enrichment analysis of the accession‐specific responsive genes reveals lipid‐remodeling as a physiological response prevalent after irradiance increase (Figure 7a). Closer analysis of these “lipid‐remodeling” irradiance responsive genes reveals that especially genes involved in phosphate‐dependent lipid‐remodeling are accession‐specifically up‐regulated upon the irradiance increase (Figures 4 and 5). Phosphate is an essential cofactor for photosynthesis and after an irradiance increase there is an increase in the pool size of phosphorylated intermediates accompanying the increased assimilation rate, requiring that more inorganic phosphate (Pi) be made available (Badger, Sharkey, & von Caemmerer, 1984; Caemmerer & Edmondson, 1986; Sivak & Walker, 1988; Walker & Sivak, 1986). A decrease in Pi such as occurs in end‐product (or sink) limitation of photosynthesis, is associated with an overall decrease in assimilation rate (Paul & Foyer, 2001; Williams, 1998). Overall, therefore, photosynthesis, cytosolic sugar metabolism, and phosphate are interconnected (Stitt, 1991). In addition, phosphate deficiency responsive genes are shown to be sugar inducible (Müller, Morant, Jarmer, Nilsson, & Hamborg Nielsen, 2007). This transcriptional correlation is thought to be important for either (a) maintenance of the ratio of available Pi and carbon by reducing the cellular sucrose content via galactolipid synthesis; or (b) for the supply of galactolipids as components of the plasma membrane to support enhance growth under sucrose supplementation (Murakawa et al., 2014). Neither Müller et al. (2007) nor Murakawa et al. (2014) have found a link between increased irradiance, photosynthetic acclimation, and phosphate deficiency. This study implicates that increased expression of phosphate‐deficiency‐initiated lipid‐remodeling genes (for the synthesis of galactolipids) in the response to an irradiance increase is a way to provide the extra Pi needed for the up‐regulation of photosynthetic and downstream carbohydrate (sucrose) metabolism (Figures 4 and 5). When comparing the three accessions (Figure 4b) the lipid‐remodeling genes are most up‐regulated in Col‐0, suggesting genetic variation in this response, associated with the variation in photosynthetic efficiency response to increased irradiance. An earlier genome‐wide association study (GWAS) among 344 Arabidopsis accession for the photosynthetic response to the same increase in irradiance confirms a role for lipid‐remodeling in photosynthetic acclimation, as the GWAS associated genomic variation in *DGD1 SUPRESSOR 1* (*DGS1*; At5 g12290) with phenotypic variation in the photosynthetic efficiency response (van Rooijen et al., 2017). *DGS1* encodes a mitochondrial outer membrane protein involved in galactolipid biosynthesis. The expression of *DGS1* is not responsive to increased irradiance, suggesting that the genomic association found in the GWAS acts on the post‐transcriptional level rather than on the transcriptional level. However, the fact that GWAS identifies a gene that is involved in phosphate‐dependent lipid remodeling strengthens the observation that variation in lipid‐remodeling genes (either genomic or expression‐based) causes natural variation in the photosynthesis efficiency response to increased irradiance.

One differentially up‐regulated lipid‐remodeling gene in the present study is *SPX1*, characterized before to be involved in phosphate deficiency response and lipid‐remodeling (Puga et al., 2014). SPX1 shares the SPX domain with yeast inorganic phosphate (Pi) sensors and plant Pi starvation signaling proteins, and is a phosphate‐dependent inhibitor of the PHOSPHATE STARVATION RESPONSE 1 (PHR1) transcription factor (Puga et al., 2014). *SPX1* is functionally redundant with *SPX2*, also found to be accession‐specifically up‐regulated in this study (Figure 4). The physical interaction between SPX1/SPX2 and PHR1 is reduced in the absence of inorganic phosphate (Pi), leading to transcription of many Pi‐starvation induced genes by PHR1. As *SPX1/2* expression itself depends on PHR1, the SPX1/2‐PHR1 interaction results in a regulatory negative feedback loop (Bustos et al., 2010; Puga et al., 2014). This means the increased expression of *SPX1/2* we observe is likely to be the consequence of a release of activated PHR1 protein, which can then induce the expression of Pi‐starvation responsive genes, including the build‐up of the negative regulator SPX1/2 itself to modulate the expression of the Pi‐starvation genes again upon increased Pi availability. Initially transcription of *PHR1* itself would not need to change as long as sufficient PHR1 is activated to initiate the Pi‐starvation response. *SPX1* is up‐regulated in Col‐0 and, to a lesser extent, in Ga‐0, but not in Ts‐1, 1 hr after the irradiance increase, and not in response to the temperature increase (Figure 6g). Rossel et al. (2002), who exposed Col‐0 plants to increased irradiance filtered to reduce the near‐infrared irradiation, also noticed the induction of *SPX1*, providing additional evidence that this gene, and the Pi‐response pathway it controls, is involved in the response to increase irradiation and not the parallel temperature increase. This is consistent with the proposed role of phospholipid‐remodeling to provide additional Pi to accommodate increased photosynthesis and sugar assimilation synthesis. For a subset of the genotypes used in this study an irradiance of 550 μmol m^−2^ s^−1^ is just saturating (van Rooijen et al., 2015) and the experiments were conducted in normal air. Given that plants used were not cold‐stressed or otherwise treated in a way that would lead to sink‐limitation of photosynthesis, it is unexpected that they would develop phosphate limitation during the experiment – this is normally only encountered as triose‐phosphate limited photosynthesis at high irradiances and carbon dioxide concentrations during, for example, A/Ci curves (Yang, Preiser, Li, Weise, & Sharkey, 2016). It seems possible, therefore, that the activation of metabolism that releases Pi might be more of a pre‐emptive response for the prospective increase in assimilation that will result from acclimation to increased irradiance. Besides *SPX1* and *SPX2*, other lipid‐remodeling genes found in this study to be responsive to increased irradiance have also been found by Puga et al. (2014), who compared the transcriptome of Pi‐deficiency treated plants with that of control plants. Among these overlapping genes are *GDPD1*,* SQD2,* and *MGD2*, involved in galactolipid biosynthesis (Figure 4). This suggests that the increased irradiance causes an as yet unidentified Pi‐limitation in the plant, that can be considered as a preparation for the increase in assimilation for what will develop over the coming days (van Rooijen et al., 2015). Considering the differential expression of these genes between the three accessions it is likely that this preparation contributes to the phenotypic variation we observed in the response to increased irradiance in Arabidopsis (Figure 1a). The response of lipid‐remodeling genes to increased irradiance led us to propose a new sensing mechanism and signal transduction pathway leading to photosynthetic acclimation response, in addition to the already described pathways (Figure 7).

### 4.2 The lipid‐remodeling pathway

Three lipid‐remodeling pathways are known to release of Pi from phospholipids during Pi deficiency (Ruelland et al., 2015). Two of these, phospholipid‐remodeling into galactolipids through the generation of phosphatidic acid (PA) by phospholipase C (first route) or phospholipase D (second route), have been extensively studied in connection to phosphate deficiency responses (Gaude, Nakamura, Scheible, Ohta, & Dörmann, 2008; Nakamura et al., 2009). We do not find either of them to respond to increased irradiance, although they are known to repress expression of the transcription factor *DREB2A* (Figure 7; (Ruelland, Djafi, & Zachowski, 2013)). The activation of the GDPD pathway (third route), as a result of Pi limitation, probably outcompetes the PLC pathway, leading to reduced PLC‐mediated repression of *DREB2A*. The third route is mediated through *GLYCEROPHOSPHO‐DIESTER‐PHOSPHO‐DIESTERASE1* (*GDPD1*), a gene known to become expressed in response to phosphate limitation (Cheng et al., 2011). The promoters of all *GDPD* genes contain Pi deficiency response elements, recognized by the PHR1 transcription factor, to initiate transcription (Cheng et al., 2011). The *GDPD* family in Arabidopsis consists of 13 genes, of which *GDPD1* is the major one involved in hydrolyzing glycerophosphodiesters during Pi starvation (Cheng et al., 2011). In all accessions we found *GDPD1* to be up‐regulated upon increased irradiance at 1 hr after the switch to an increased irradiance (Figures 4b and 5b). To release Pi from phospholipids, the phospholipids are first converted into glycerophophodiesters and then hydrolyzed into glycerol‐3‐phosphate (G‐3‐P) by glycerophospho‐diester‐phospho‐diesterase GDPD1. It is unknown how Pi is released from G‐3‐P in Pi‐starved plants, but this most likely occurs via the de novo synthesis of digalactosyl diacylglycerol (DGDG) and sulfoquinovosyl diacylglycerol (SQDG) (Cheng et al., 2011). In this pathway, G‐3‐P is first converted into phosphatidic acid (PA) and Pi is released in the subsequent conversion of PA into diacylglycerol (DAG) by phosphatidic acid phosphohydrolase (PAH), (Nakamura et al., 2009). DAG is the direct substrate for synthesis of either monogalactosyl diacylglycerol (MGDG; by MONOGALACTOSYLDIACYLGLYCEROL SYNTHASE 1 (MGD1)*,* MGD2 and MGD3), digalactosyl diacylglycerol (DGDG; by DIGALACTOSYL DIACYLGLYCEROL SYNTHASE 1 (DGD1) and DGD2), or SULFOQUINOVOSYL DIACYLGLYCEROL (SQDG; by SULFOQUINOVOSYL DIACYLGLYCEROL SYNTHASE 1 (SQD1) and SQD2), where MGDG is the precursor of both DGDG and SQDG (Figure 7b). MGDG, DGDG, and SQDG are bilayer‐forming galactolipids, important for the integrity of the photosynthetic membrane and of the chloroplast protein‐import apparatus (Chen & Li, 1998; Kansy, Wilhelm, & Goss, 2014). DGDG is known to replace phospholipids in membranes in phosphate‐limited conditions (Härtel, Dörmann, & Benning, 2000). Mutation of the *GDPD1* gene has no effect on the DGDG content, and the effect on the SQDG content is not known (Cheng et al., 2011). We do not observe induced transcription of *DGD1* and *DGD2* in response to the increase in irradiance, but transcription of both *SQD1* and *SQD2* is up‐regulated in Col‐0, comparable to the induction of *SPX1* and *GDPD1* (Figures 4b and 5b). This suggests that at least part of the release of Pi, which is needed to deal the incipient phosphate‐limited conditions at increased irradiance, is mediated through SQDG synthesis.

The expression of lipid‐remodeling genes, normally regulated by the SPX1/2‐controlled PHR1 transcription factor, are induced within 1 hr after irradiance increase. We notice an interesting expression pattern for another transcription factor gene known to have a role in photosynthesis: *GOLDEN2‐LIKE 2* (*GLK2*). Transcription of *GLK2* is down‐regulated at 1 hr after the irradiance increase, but is induced after 3.5 hr (Figure 6). Golden2‐like transcription factors (GLK) regulate nuclear genes involved in chlorophyll biosynthesis and are required for the development of photosynthetic proteins in chloroplasts (Chen et al., 2016; Wang et al., 2017; Waters et al., 2009). GLK2 is also a pivotal regulator of *DGD1* transcription, which encodes the key enzyme for DGDG synthesis (Kobayashi et al., 2014). The expression profile of *GLK2* (Figure 6c) implies that GLK2‐induced DGDG replacement of phospholipids is activated only after 3.5 hr of excess light, following the initial induction of *SPX1/2* and *GDPD1*, after 1 hr. We therefore hypothesize that the preference for SQDG (over DGDG) for lipid‐remodeling of photosynthetic membranes in response to irradiance increase is mediated by reduced DGDG synthesis as result of decreased *GLK2* expression.

### 4.3 Expression variation in heat‐shock factors associates with natural variation in photosynthesis response to increased irradiance

The core set of transcriptionally responsive genes is enriched for genes involved in photosynthesis, but also for heat‐shock response and signaling through transcriptional response (“RNA binding”; Figure 3a). Leaf temperature is the result of the balance of different heat flows to and from the leaf (Nobel, 2005). An increase in irradiance represents an increase in the short‐wave radiation flow to the leaf which tends to increase leaf temperature, but the increase in assimilation it produces results in an increase in stomatal conductance and thus transpiration, resulting in a latent heat flux from the leaf which tends to cool it. Whether the warming or the cooling effect associated with an increase in photosynthetically active irradiance will dominate will depend on the spectral distribution of the radiation, the boundary layer and stomatal conductances, and the vapor pressure deficit between the leaf air space and the mixed air around the leaf. In a controlled environment room with limited air movement (and thus a low boundary layer conductance) a temperature increase will nearly always parallel an irradiance increase, and temperature changes produce their own transcriptome changes. In the field, however, transpirative cooling can lower leaf temperature below air temperature (Larcher, 1995). The transcriptome changes provoked by an irradiance increase can actually be due to the temperature increase rather than the photochemical (photosynthetic and photoreceptor) changes resulting from a higher irradiance (Swindell et al., 2007), explaining the core responsiveness of heat‐shock factors.

It is possible to separate the photosynthetic and photoreceptor‐based effects of an irradiance increase from the temperature‐based effect. This can be achieved by using heat filters to block the (near) infrared light that is responsible for part of the temperature increase (Rossel et al., 2002), or by running parallel experiments with an irradiance increase and an accompanying temperature increase alongside a temperature increase only. In this study, we use the latter approach and find that the induction of expression of several heat‐shock response genes is mostly heat responsive and not specific for the irradiance increase, as they are also induced upon a temperature increase only. The expression of the *HSFA2* and *HOP3* genes is determined in more detail (Figure 6d,e). *HSF2A* encodes a Heat‐shock transcription factor associated with photosynthesis acclimation through the ascorbate peroxidase pathway (Jung et al., 2013; Nishizawa et al., 2006). *HOP3* (*HSP70‐HSP90 ORGANIZING PROTEIN3*), encodes a tetratricopeptide repeat protein that acts as a chaperone in the interaction with HSP70 and HSP90 to assist in the import of photosynthetic pre‐proteins synthesized outside the chloroplast. It also regulates the stress response of the endoplasmic reticulum (Fellerer, Schweiger, Schöngruber, Soll, & Schwenkert, 2011; Fernández‐Bautista, Fernández‐Calvino, Muñoz, & Castellano, 2017). Both *HSFA2* and *HOP3* are induced in Ga‐0 and Col‐0, but only at 1 hr, and no longer at 3.5 hr, after the increase in irradiance and they are induced in all three accessions after the increase in temperature (Figure 6). Although out of all induced heat response genes (Figures 3, 4, 5) we only confirm the heat‐induced transcriptional response for these two known heat response associated genes (Figure 6), it is likely to be the general pattern that their induction of expression is mainly in response to the increase in temperature that in our case accompanied the increase in irradiance (note that an increase in irradiance can result in leaf cooling due to the increase in transpiration). This does not mean that this response is unimportant for the acclimation to increased irradiance if this is accompanied by a temperature increase. In this respect it is important to note that the strong induction of heat‐shock response genes in Col‐0 and Ga‐0 in response to increase in irradiance is not seen in Ts‐1, which correlates well with the differences in photosynthesis efficiency between Ts‐1, Col‐0 and Ga‐0 (Figure 1a). These observations lead us to conclude that the expression response of heat‐shock genes might influence the degree in which photosynthetic light‐use efficiency is affected in response to increased irradiance, and which, on longer time scale, might also affect the photosynthetic acclimation.

### 4.4 Expression variation in ERF/AP2 transcription factors associates with natural variation in photosynthesis response to increased irradiance

Looking further among the core responsive genes, several members of the ERF/AP2 transcription factor family are found. The ERF/AP2 transcription factor family is known to sense and signal the response to changed metabolite levels resulting from increased irradiance levels; members of this family have been found before as regulators of early downstream targets in the acclimation response to high light (Alsharafa et al., 2014; Moore, Vogel, & Dietz, 2014). They are induced by the transcription factor ZAT10 (Alsharafa et al., 2014; Nguyen et al., 2012), which functions to initiate a systemic acclimation response to excess light (Munekage, Inoue, Yoneda, & Yokota, 2015; Rossel et al., 2007). *ZAT10* transcription is also up‐regulated in our study (Figure 5b).

Among the irradiance responsive ERF/AP2 TFs, *DREB2A* stands out because its degree of up‐regulation is accession‐specific but not time‐specific, and its expression correlates (negatively) with the variation in photosynthetic efficiency between the accessions (Supporting Information Tables S1 and S2). The expression of *DREB2A* is specifically responsive to increased irradiance and not to increased temperature (Figure 6). *DREB2A* encodes a transcription factor belonging to the ERF/AP2‐type transcription factor family, binding to drought responsive elements (DREs) in the promoters of transcriptional target genes. *DREB2A* is known as a key regulator of drought response (Sakuma, Maruyama, Osakabe, et al., 2006), though it is also known to be activated in response to heat stress (Sakuma, Maruyama, Qin, et al., 2006), as well as increased irradiance (Rossel et al., 2002). *DREB2A* is transcriptionally up‐regulated 1 hr after irradiance increase, only in Col‐0 (Figure 6i), suggesting an accession‐specific involvement of DREB2A in regulating the photosynthesis response to irradiance increase in Arabidopsis, probably through the SPX1/PHR1‐mediated response pathway acting on lipid remodeling (Figure 7b). DREB2A is not the only ERF/AP2 transcription factor of which its expression correlates with photosynthetic efficiency response; also *ERF53* correlated, although in positive direction (Supporting Information Table S3). ERF53 is heat responsive and regulates stomatal movement (Hsieh, Cheng, & Lin, 2013).

### 4.5 Expression variation in alternative splicing factors associates with natural variation in photosynthesis response to increased irradiance

Another core responsive gene for which the degree of up‐regulation is accession‐specific, but not time‐specific, and for which the expression correlates (positively) with the variation in photosynthetic efficiency encodes the alternative splicing factor SR45 (Supporting Information Tables S1 and S3). SR45a is a protein involved in stress‐induced alternative splicing (Sakuma, Maruyama, Qin, et al., 2006; Yoshimura et al., 2011). Expression of *SR45A* is responsive to both heat and light (Figure 6). Not only *SR45A*, but also another serine/arginine‐rich protein (SR) gene, *SR30*, is found to be up‐regulated in response to increased irradiance, and both respond most strongly 3.5 hr after the irradiance increase (Figure 5). Both genes were initially classified as members of the serine/arginine‐rich protein family (Tanabe, Yoshimura, Kimura, Yabuta, & Shigeoka, 2007), however, a rational and consistent nomenclature for SR protein splicing factors excluded SR45a from the serine/arginine rich protein family, based on its sufficiently different protein sequence (Barta, Kalyna, & Reddy, 2010). Nevertheless, despite the sequence differences, later experimentation proved SR45a to function in stress‐induced alternative splicing (Yoshimura et al., 2011). Both *SR30* and *SR45a* are up‐regulated in response to high light stress (Rossel et al., 2002; Tanabe et al., 2007). No obvious aberrant phenotype has been observed for the *sr45a* mutant (Yoshimura et al., 2011). The *SR30* gene has so far not been investigated in great detail, but the similarity in gene expression profile (Figure 6b) and the similarity in subcellular localization (Mori et al., 2012) suggest that *SR45A* and *SR30* cooperate in the regulation of alternative splicing. The micro array we have used for transcript profiling does not allow the detection of alternative splicing, but expression of these genes suggests this may be affected by the increased irradiance.

## 5 CONCLUSIONS

We identify a phospholipid‐remodeling pathway as important for the acclimation to increased irradiance in Arabidopsis. This pathway starts with the SPX1‐mediated activation of the transcription factor PHR1 leading to the transcription of *GDPD1*, enhancing galactolipid biosynthesis and eventually leading to DGDG and SQDG synthesis (Figure 7b), important for the lipid‐remodeling of the photosynthetic membrane. Although the involvement of PHR1 in the response to high light had been noted (Nilsson, Lundmark, Jensen, & Nielsen, 2011), information on which subsequent steps might be involved was still lacking. We propose it involves the substitution of phospholipids by galactolipids through SQDG (preferred over DGDG) to release Pi needed for acclimating photosynthesis rates.

In addition to the up‐regulated expression of phospholipid‐remodeling pathway, we identify the up‐regulation of *SR45A* and *SR30* transcription in response to irradiance increase. These genes are involved in mediation of alternative splicing, suggesting alternative transcripts are important for the acclimation to increased irradiance, but the targets of these regulators and their roles in the acclimation process remain to be elucidated.

It is known that retrograde signaling is light intensity‐dependent, as being the prevalent sensory pathway in excess light providing a mechanism for protection against photo‐oxidative damage by suppressing photomorphogenic development and minimizing tissue exposure to damaging irradiance (Martin et al., 2016; Szechynska‐Hebda & Karpinski, 2013). We have shown this correlates with natural variation in photosynthetic response to high light as in this study the lower expression of cell growth regulatory genes positively correlated with Ф_PSII_ in the different accessions (Figure 4). In addition, we have shown the correlation of carbohydrate metabolism with natural variation as the gene ontology term enriched among the accessions specific up‐regulated genes (Figure 4), shown before to act as a short‐term response to high irradiance exposure (Schmitz et al., 2014).

In this study, one of the expression pathways that correlate with the observed phenotypic differences in acclimation between three Arabidopsis accessions is the up‐regulation of *SR45A* and *SR30*. Second, stronger expression of heat responsive genes in the accession Ga‐0 might explain its slightly higher photosynthesis efficiency compared to Col‐0, while the absence of induced expression of heat responsive genes in Ts‐1 could partly explain its lower photosynthetic efficiency. Third, the PHR‐mediated gene activation pathway, that was found in this study to play a role in response to increased irradiance, was found to be active in both Ga‐0 and Col‐0, providing an additional explanation for the higher photosynthesis efficiencies in these accessions compared to Ts‐1, in which the activation of both the heat‐shock responsive pathway as well as the PHR‐mediated pathway is absent. Fourth, we found a time‐specific expression response of genes involved in flavonoid/anthocyanin biosynthesis in Col‐0, correlating with a fast rise in anthocyanin content in Col‐0, compared to Ts‐1 and Ga‐0, most likely involved in scavenging of ROS. As a last observation, transcription factors belonging to the ERF/AP2 family were found to be responsive to increased irradiance in all three accessions, but the specific genes of this family and the direction of the response differs between the three accessions. All in all, this study shows that natural variation for a complex physiological response such as photosynthetic efficiency response to increased irradiance, correlates to variation in transcriptional response of variable genes and processes. To identify the exact alleles that encode this variation will be the next challenge.

## AUTHORS’ CONTRIBUTIONS

All authors designed the study, RvR performed the experiments and analyzed the data, all authors wrote the manuscript.
