# Supplementary material for: Photosynthetic response to increased irradiance correlates to variation in transcriptional response of lipid‐remodeling and heat‐shock genes
Source: Plant Direct. 2018 Jul 10;2(7):e00069. doi: 10.1002/pld3.69 (PMC6508758; doi:10.1002/pld3.69)
Supplement: Supplementary file 1 [file PLD3-2-e00069-s001.pdf]

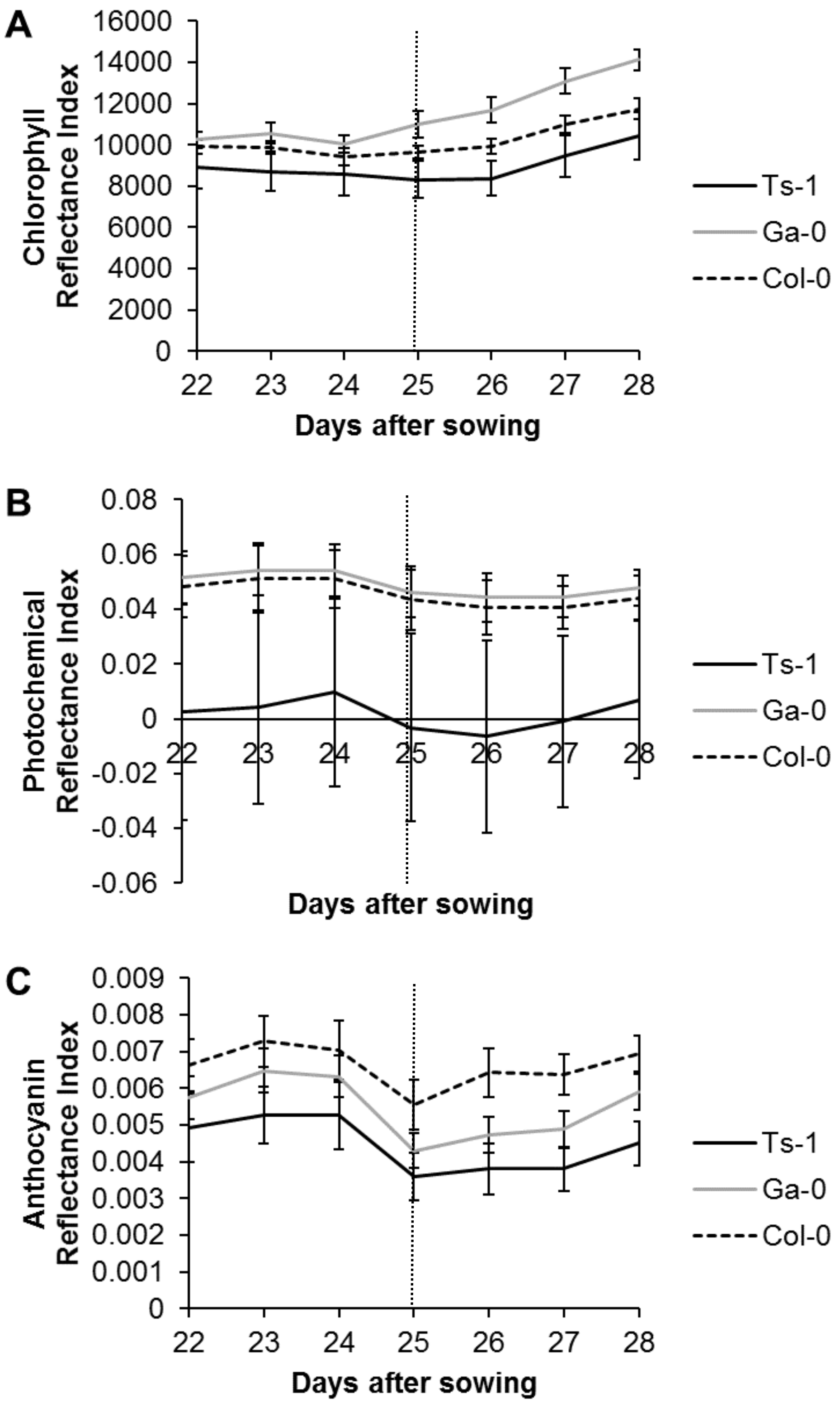

Figure S1. The effect of genotype on chlorophyll, photochemical, and anthocyanin reflectance indices over time in response to increased irradiance in Arabidopsis. Representative phenotypes of (A) chlorophyll reflectance index, (B) photochemical reflectance index, and (C) anthocyanin reflectance index for Arabidopsis accessions Col-0, Ga-0 and Ts-1, grown for 24 days in 100 µmol m^{-2} s^{-1} growth irradiance and subsequently 6 days in 550 µmol m^{-2} s^{-1} growth irradiance, measured from day 23 until day 30 after sowing, at four time-points per day.
