# Supplementary material for: Photosynthetic response to increased irradiance correlates to variation in transcriptional response of lipid‐remodeling and heat‐shock genes
Source: Plant Direct. 2018 Jul 10;2(7):e00069. doi: 10.1002/pld3.69 (PMC6508758; doi:10.1002/pld3.69)
Supplement: Supplementary file 3 [file PLD3-2-e00069-s003.pdf]

Dear Dr. Ana Fortes,

Thank you for your last suggestion regarding our manuscript. We have removed the detailed results from the conclusive paragraph in the discussion to the more appropriate places in the manuscript, and we hope with that we have fulfilled all criteria for publication.

Yours sincerely,

Roxanne van Rooijen
Jeremy Harbinson
Mark Aarts
